# Lung Nodule and Food Bolus Impaction: Can They Be Related?

**DOI:** 10.7759/cureus.12351

**Published:** 2020-12-29

**Authors:** Lucia Carvalho, Marta Guimarães, Ana Marta Pereira, Rui F Almeida, Mário Nora

**Affiliations:** 1 General Surgery, Centro Hospitalar de Entre o Douro e Vouga, Santa Maria da Feira, PRT

**Keywords:** achalasia, esophageal motor disorder, achalasia cardia, esophageal myotomy, heller myotomy, heller's esophagus-cardiomyotomy, recurrent aspiration pneumonia

## Abstract

Achalasia is a rare primary disorder of esophageal motility characterized by insufficient lower esophageal sphincter relaxation and loss of esophageal peristalsis. This results in patient complaints of dysphagia to solids and liquids, regurgitation, chest pain, and weight loss. However, achalasia may also present with respiratory symptoms, such as aspiration pneumonia, due to remarkable regurgitation. In untreated patients and a long period of evolution, respiratory symptoms may even be the initial manifestation of achalasia. An endoscopic finding of retained food and saliva with a puckered gastroesophageal junction or barium swallow showing dilated esophagus with birds beaking in a symptomatic patient should prompt appropriate diagnostic and therapeutic strategies. We describe an atypical presentation of a rare disease in a young man with a history of symptoms caused by the late manifestation of achalasia.

## Introduction

Achalasia is the best-defined primary motor disorder of the esophagus, characterized by a failure of the lower esophageal sphincter (LES) to relax during swallowing, often combined with a hypertensive muscular tone [1]. This leads to a loss of the peristaltic abilities of the esophageal corpus and obstruction of the bolus transport. Patients present with dysphagia to solids and liquids, regurgitation, halitosis, and, occasionally, chest pain with or without weight loss [2]. However, achalasia may also present with respiratory symptoms that could mimic other pulmonary disorders and delay the proper diagnosis of achalasia [3].

Diffuse aspiration bronchiolitis caused by recurrent aspiration is an entity that is described as associated with achalasia due to nocturnal regurgitation [4]. Upper endoscopy and esophagography with barium swallow should be done to rule out pseudoachalasia - mechanical reasons for dysphagia such as malignant disease, scarring, or peptic stricture. An endoscopic finding of retained food and saliva with a puckered gastroesophageal junction or barium swallow showing dilated esophagus with ‘bird’s beak’ appearance are typical findings although the definitive diagnosis of achalasia is made through high-resolution esophageal manometry [5]. Achalasia treatment with improvement in regurgitation should also lead to the resolution of respiratory complications.

We present a case of incidental diagnosis of achalasia identified during the study of a pulmonary nodule, associated with significant weight loss and food intolerance.

## Case presentation

A 34-year-old male with no relevant medical history presented to the emergency department for an inability to swallow, with food and saliva regurgitation after having eaten bread. For suspected food impaction, an upper endoscopy was performed. The finding was: “Esophagus: lumen filled with bulky food cake, which makes it difficult for the device pass to the cardia.”

**Figure 1:**
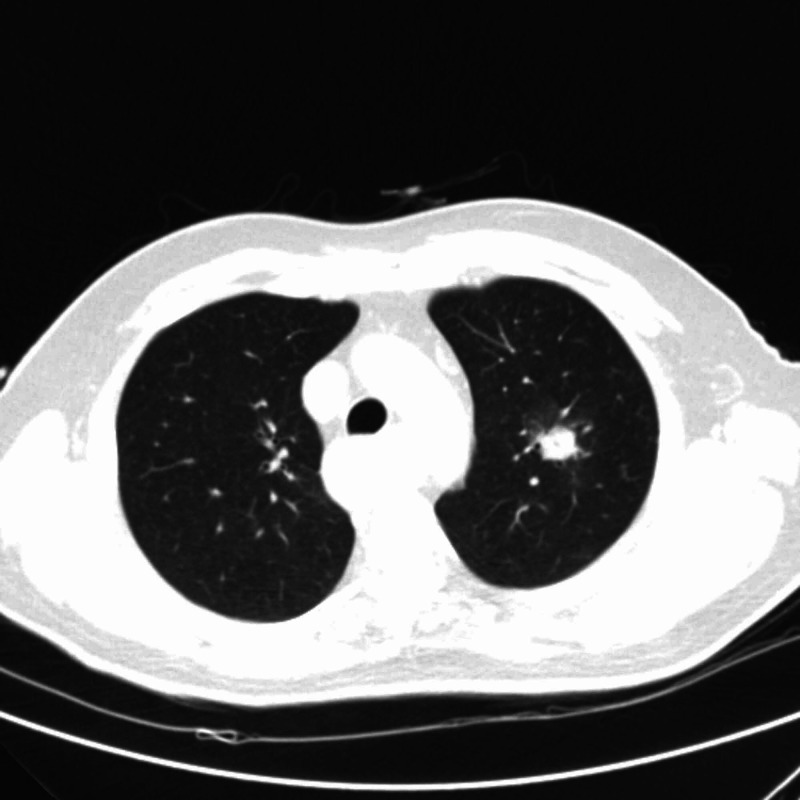
Chest CT scan Presence of a spiky, solid image in the upper left lobe, with 22 mm diameter and ground-glass opacity, highly suggestive of a neoplastic process. CT: computed tomography

**Figure 2:**
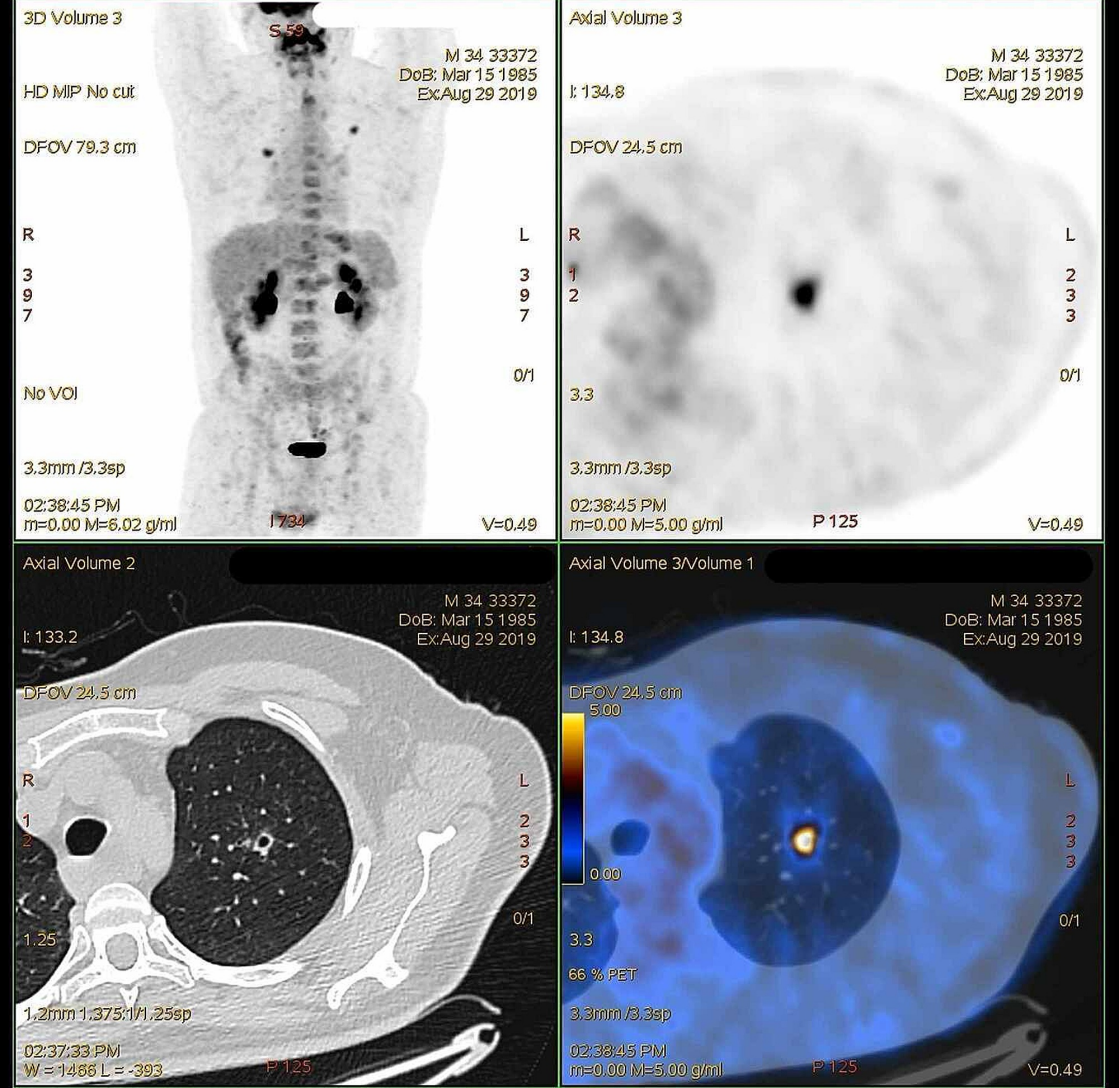
PET-CT Identification of a hypermetabolic nodule in the upper left lobe, with irregular contours, cavitated, measuring about 16 mm. There are also several nodules in the left lung, with limited functional characterization due to their small dimensions. PET-CT: positron emission tomography-computed tomography

The patient complained of regurgitation with about three months evolving, mostly for liquids, and non-productive cough. He denied dysphagia or chest pain. He also reported weight loss of about 40 kg in a month and a half. He was not a smoker, and his family medical history was unremarkable. On physical examination, he had fever and rhonchi on pulmonary auscultation in the left hemithorax. Chest computed tomography (CT) scan showed a spiky, solid image in the upper left lobe, highly suggestive of a neoplastic process (Figure 1). Positron emission tomography-computed tomography (PET-CT) presented a spiculated formation of 16.2 mm corresponding to a focus of intense fluorodeoxyglucose (FDG) uptake, according to neoplasia (Figure 2). Tumor markers were within the normal range, and pulmonary biopsy was negative for neoplastic cells.

**Figure 3:**
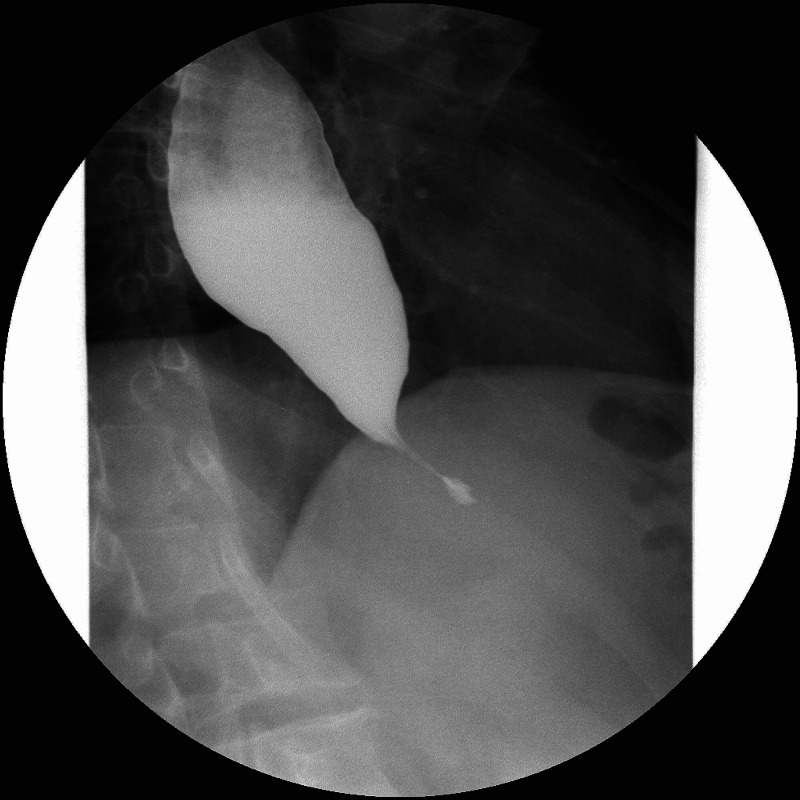
Contrast esophagram Upper gastrointestinal tract radiography with barium contrast showed a dilated esophagus with a tapered narrowing of the lower end – bird's beak appearance

He repeated upper endoscopy after the acute episode of food impaction; there was difficulty in transposing the cardia with resistance to transposition but without evident injuries. Both chest CT and PET-CT also showed marked dilatation of the esophagus along its entire length without significant parietal thickening, namely in the terminal portion in the cardia. The barium esophagram showed a dilated esophagus with a tapered narrowing of the lower end - bird's beak appearance (Figure 3). The diagnosis of achalasia type II was made with high-resolution manometry. In this case (a non-smoker, young male without comorbidities or family history of cancer), the diagnosis of a pulmonary tumor would be less likely, which led to discussing the case with radiologists who identified other small and round pulmonary opacities, diffusely spread mainly in a bronchocentric localization, which raised the hypothesis of aspiration pneumonia.

The patient underwent antibiotic treatment for 14 days, leading to fever disappearance and gradual reduction of the left pulmonary nodular image. During this period, the patients' condition improved, and he was discharged to complete antibiotherapy treatment in ambulatory, but food intolerance led him to another hospital admission when he completed antibiotic treatment. He repeated chest CT that showed almost complete resolution of pulmonary nodules.

After clinical and imaging resolution, he underwent Heller's esophagus cardiomyotomy by laparoscopy combined with Dor fundoplication. The patient recovered uneventfully. He is asymptomatic six months after surgery, and he gained 22 kg.

## Discussion

Achalasia is a rare primary motor disorder of the esophagus characterized by insufficient LES relaxation and loss of esophageal peristalsis [6]. Typically, achalasia affects both genders equally and is frequently diagnosed in patients aged between 40 and 60 years [7]. The transit of swallowed food boluses through the esophagus is impaired, and the patient classically presents with dysphagia to both solids and liquids. The regurgitation of saliva and undi­gested food can cause heartburn, retrosternal or burning pain, weight loss, and dyspepsia, which often leads to a misdiagnosis of gastroesophageal reflux disease [8]. Further associated manifestations involve respiratory symptoms such as nocturnal cough, recurrent breathing difficulties, and even pneumonia [9]. In patients with respiratory manifestations, the initial management could often be directed to pulmonary work-out. Nevertheless, these symptoms may actually arise from pathological processes that involve non-pulmonary thoracic organs [10]. Though, it is important to keep in mind that esophageal diseases like achalasia may mimic respiratory conditions since nocturnal aspiration could cause recurrent aspiration pneumonitis as reported in the literature in almost 10% of all untreated achalasia patients [11].

Achalasia is difficult to diagnose early but it is important to promptly identify and treat this condition before irreversible changes occur. This is a progressive disease that may advance to megaesophagus if left untreated. Moreover, it is also with an increased risk of esophageal squamous cell carcinoma [12]. Various diagnostic modalities have been implemented to get the diagnosis. The gold standard is high-resolution manometry, which not only establishes the diagnosis but also classifies the achalasia subtypes that are used to predict response to medical or surgical therapy [13]. Upper endoscopy is indicated to rule out mimickers of the disease known as pseudoachalasia. The endoscopic appearance of a dilated esophagus with retained food or saliva and a puckered lower esophageal sphincter in the absence of attributing strictures or tumors should raise suspicion for achalasia [14]. Additionally, a barium esophagogram may reveal poor emptying of barium, esophageal dilation, and minimal LES opening, resulting in a tapering of the barium column, giving it a bird’s beak appearance [15] as seen in Figure 3. Regurgitation becomes a major problem as the disease progresses and, especially, when the esophagus begins to dilate, as seen in images of this patient.

In this case, food intolerance due to regurgitation was an indicator but respiratory symptoms hindered the achalasia diagnosis. Although he was a non-smoker young male without comorbidities or family history of cancer, the presence of pulmonary nodule suggestive of malignancy in chest CT, weight loss, and respiratory symptoms initially led to the diagnosis of lung cancer. The PET-CT confirmed the presence of pulmonary nodule but also revealed a dilatated fully esophagus as present in CT images. Bronchoalveolar lavage showed the absence of neoplastic cells, and tumor markers were within the normal range. These results, associated with the patient's complaints and endoscopy findings, led to a diagnosis of achalasia diagnosis, confirmed by manometry. A multidisciplinary discussion with radiologists and pneumologists was essential in this case to achieve the diagnosis of aspiration pneumonia that was much more likely in this setting than lung cancer. Although a pulmonary biopsy could also help, it was not performed as the nodule decreased after antibiotherapy implementation, confirming the non-neoplastic diagnosis. Nutritional support is likewise an important concern in achalasia patients that present with long-term food intolerance and significant weight loss so this patient required parenteral nutrition before surgical treatment.

Though no current treatment option is a definitive cure for achalasia, the aim of the different treatments is to reduce the resistance to esophageal emptying by reducing LES hypertonicity as meaningful peristaltic activity cannot be restored by any intervention [16]. The purpose of all treatment modalities is to relieve symptoms, improve esophageal emptying, and prevent complications such as weight loss, aspiration pneumonia, and further esophageal dilation [17]. This is attempted via pharmacologic, endoscopic, or surgical means [18]. The treatment choice depends on the patient’s comorbidities, operative risk factors, disease stage, and previous treatments [19]. The standard treatment for achalasia has been a distal esophageal myotomy combined with a partial fundoplication (either Dor or Toupet), with success rates that differ according to the achalasia subtype [20]. This patient was submitted to a laparoscopic Heller myotomy with Dor fundoplication, resulting in the disappearance of dyspepsia, regurgitation, and pulmonary complications. Therefore, achalasia represents an important differential diagnosis in patients with aspiration pneumonia, particularly among the younger subjects.

## Conclusions

Achalasia is a rare primary motor disorder of the esophagus characterized by patient complaints of dysphagia to solids and liquids, regurgitation, and halitosis. Occasionally, chest pain, weight loss, and respiratory symptoms caused by regurgitation could also be present. We describe an atypical presentation of achalasia in a young man with a history of pulmonary manifestations of regurgitation. In the absence of suspicion of achalasia, a patient may initially be managed for respiratory disorders, which may lead to a diagnostic delay. Diagnosis of achalasia should be kept in mind in patients with respiratory symptoms, particularly in the differential diagnosis of younger patients who are less likely to have such conditions. A close collaboration between the radiologist, gastroenterologist, and surgeon in evaluating this complex group of patients is essential for a correct diagnosis. This case also emphasizes the importance of a carefully recorded history, including the patient's medical history and habits and the selection of tests performed to diagnose esophageal function disorders that can mimic other conditions.

## References

[1] Jeon HH, Kim JH, Youn YH, Park H, Conklin JL (2017). Clinical characteristics of patients with untreated achalasia. J Neurogastroenterol Motil.

[2] Francis DL, Katzka DA (2010). Achalasia: update on the disease and its treatment. Gastroenterology.

[3] Pandolfino JE, Gawron AJ (2015). Achalasia: a systematic review. JAMA.

[4] Teramoto S, Yamamoto H, Yamaguchi Y, Tmoita T, Ouchi Y (2004). Diffuse aspiration bronchiolitis due to achalasia. Chest.

[5] Vaezi MF, Pandolfino JE, Vela MF (2013). ACG clinical guideline: diagnosis and management of achalasia. Am J Gastroenterol.

[6] Sadowski DC, Ackah F, Jiang B, Svenson LW (2010). Achalasia: incidence, prevalence and survival. A population-based study. Neurogastroenterol Motil.

[7] Jung HK, Hong SJ, Lee OY (2020). 2019 Seoul consensus on esophageal achalasia guidelines. J Neurogastroenterol Motil.

[8] Richter JE (2011). The diagnosis and misdiagnosis of achalasia: it does not have to be so difficult. Clin Gastroenterol Hepatol.

[9] Eckardt AJ, Eckardt VF (2009). Current clinical approach to achalasia. World J Gastroenterol.

[10] Arslan M, Diken OE, Adali GK (2016). A case of achalasia causing aspiration pneumonia. Turkiye Klin Arch Lung.

[11] Duranceau AC, Deschamps C, Lafontaine E (1991). Achalasia (hypomotility) is the best known entity. Primary Motility Disorders of the Esophagus.

[12] Gillies CL, Farrukh A, Abrams KR, Mayberry JF (2019). Risk of esophageal cancer in achalasia cardia: a meta-analysis. JGH Open.

[13] Boeckxstaens GE, Zaninotto G, Richter JE (2014). Achalasia. Lancet.

[14] Nijhuis O, Zaninotto G, Roman S (2020). European guideline on achalasia - UEG and ESNM recommendations. United Eur Gastroenterol J.

[15] Levine MS (2018). Ten questions about barium esophagography and dysphagia. Gastroenterol Clin North Am.

[16] Nurczyk K, Patti MG (2020). Surgical management of achalasia. Ann Gastroenterol Surg.

[17] Yeo CJ (2019). Shackelford’s Surgery of the Alimentary Tract, Eighth Edition.

[18] Patel DA, Lappas BM, Vaezi MF (2017). An overview of achalasia and its subtypes. Gastroenterol Hepatol.

[19] Krill JT, Naik RD, Vaezi MF (2016). Clinical management of achalasia: current state of the art. Clin Exp Gastroenterol.

[20] Cameron AM, Cameron JL (2020). Current Surgical Therapy, Thirteenth Edition.

